# Characteristics and outcomes of patients undergoing colonoscopy in Gaza Strip hospitals: a retrospective study

**DOI:** 10.1186/s12876-026-04673-0

**Published:** 2026-02-06

**Authors:** Khaled Siyam, Khamis Elessi, Mosheer Al-Dahdouh, Ahmed Eid, Hasan Hamdan, Mustafa Abu Jayyab, Issam Awadallah, Tayseer Afifi

**Affiliations:** 1 Department of General Surgery, Indonesian Hospital, Ministry of Health, Gaza, Palestine; 2 Faculty of Medicine, Islamic University of Gaza, Gaza, Palestine; 3 Department of General Surgery, Nasser Medical Complex, Ministry of Health, Gaza, Palestine; 4 Endoscopy Unit, Shifa Medical Complex, Ministry of Health, Gaza, Palestine

**Keywords:** Colonoscopy, Colorectal cancer, Gastrointestinal bleeding, Gaza strip, Retrospective study

## Abstract

**Background:**

Colonoscopy is a key diagnostic and therapeutic tool for colorectal diseases, including cancer, polyps, and bleeding disorders. In low-resource settings such as the Gaza Strip, challenges in access to healthcare may influence patient characteristics, indications, and outcomes of colonoscopy. Understanding these patterns is critical to improving diagnostic services and patient care.

**Methods:**

This retrospective analytical study was conducted at the endoscopy unit of Gaza Strip hospitals and included all patients who underwent colonoscopy between 2017 and 2022. A total of 983 patients were identified from hospital records. Data on sociodemographic characteristics, presenting complaints, colonoscopic findings, and physician recommendations were collected using a structured questionnaire. Statistical analysis was performed using SPSS v23, with descriptive and inferential methods applied.

**Results:**

Among 983 patients undergoing colonoscopy (mean age 52 ± 16 years; 61.4% male), most procedures were outpatient (93.6%) and performed under general anesthesia (97.8%). The most common indications were bleeding (35.2%) and follow-up (15.9%). Colonoscopy reached the cecum in 46.7% of cases. Findings were normal in 35.4%, while masses (11.0%), polyps (10.2%), diverticulae (5.0%), and hemorrhoids (7.5%) were the most frequent abnormalities. Abnormal findings were significantly associated with older age, male sex, non-outpatient admission, and bleeding as indication (*p* < 0.01). Management reflected procedural outcomes: normal findings mainly required follow-up, whereas significant abnormalities were more often referred for medical or surgical intervention (*p* < 0.001).

**Conclusion:**

This study highlights that colonoscopy in Gaza Strip hospitals predominantly serves middle-aged male patients, with bleeding being the most common indication. While many patients had normal findings, a considerable proportion presented with polyps, masses, or other abnormalities requiring further management. These findings underscore the importance of colonoscopy as a diagnostic and preventive tool and emphasize the need to strengthen screening and early detection strategies in resource-limited settings.

**Supplementary Information:**

The online version contains supplementary material available at 10.1186/s12876-026-04673-0.

## Introduction

Colonoscopy is a cornerstone procedure in the diagnosis, prevention, and management of colorectal diseases, including colorectal cancer (CRC), inflammatory bowel disease, and gastrointestinal bleeding. Globally, CRC represents a major public health concern, ranking as the third most common malignancy and the second leading cause of cancer-related mortality [1]. In many developed countries, the incidence of CRC has stabilized or even declined in older populations, largely due to the implementation of effective screening programs such as colonoscopy and fecal occult blood testing, which enable the early detection and removal of precancerous adenomatous polyps [2–4]. Increased public awareness regarding CRC risk factors has also contributed to improved outcomes.

Despite these advances, recent epidemiological data indicate a concerning rise in early-onset CRC (ages 20–49 years), with incidence rates increasing from 4.2 to 6.7 per 100,000 between 1990 and 2019 [5]. Younger patients are often diagnosed at more advanced stages compared to older individuals, which negatively influences prognosis [6]– [7]. Studies utilizing the Surveillance, Epidemiology, and End Results (SEER) database have shown a significantly higher proportion of stage III and IV disease in younger patients than in their older counterparts [8].

Outcomes of patients undergoing colonoscopy vary widely depending on demographic factors, clinical presentation, and healthcare system capacity. In the Middle East, including Saudi Arabia, CRC represents a growing burden. It is the most common cancer among men (13%) and the second most common among women (9%), with nearly 1,200 cases reported in 2011 [9]. Notably, the mean age of CRC diagnosis in the region (55–58 years) is approximately 12–15 years younger than in Western populations [10–12]. Although the overall age-standardized incidence of CRC remains lower than that of Western countries, the rates are rising rapidly, largely attributed to lifestyle changes and dietary Westernization [13–15].

In the Gaza Strip, limited resources, restricted access to healthcare, and ongoing political and economic challenges significantly constrain endoscopic capacity. These limitations reduce opportunities for population-based screening colonoscopies, leading to a predominance of symptomatic rather than preventive procedures. Consequently, most colonoscopies are performed for urgent indications such as bleeding or abdominal pain, while routine screening remains exceptionally rare. This combination of high morbidity, limited procedural capacity, and a relatively low proportion of benign findings highlights the unique clinical significance of colonoscopy outcomes in this context. Understanding these patterns is crucial for guiding clinical practice, informing public health strategies, and strengthening early detection efforts in resource-constrained environments.

This study focuses specifically on endoscopic outcomes, including procedural findings, technical completeness, and immediate clinical recommendations, as the retrospective nature of the available records and fragmented follow-up data do not allow reliable assessment of long-term patient health outcomes. Consequently, the findings are expected to reflect the predominant pattern of colonoscopy practice and diagnostic yield within the public healthcare system in Gaza. The present study aimed to evaluate the characteristics and outcomes of patients undergoing colonoscopy in Gaza Strip hospitals through a retrospective analysis.

## Methods

### Study design

This research employed a retrospective descriptive study design. The retrospective approach was chosen as it allows the analysis of existing medical records to identify patterns and associations without influencing patient care or outcomes. The descriptive component was necessary to outline the demographic and clinical characteristics of patients who underwent colonoscopy. Given that the primary objective of the study was to assess the clinical indications, presenting features, and outcomes of colonoscopy among patients at the Gaza Strip Hospitals, this design was deemed the most appropriate and methodologically sound.

### Study setting

The study was carried out at the endoscopy unit of the Gaza Strip Hospitals (Shifa Medical Complex, Nasser Medical Complex and European Gaza Hospital) The hospitals’ endoscopy unit is well-equipped with modern diagnostic tools and receives referrals from various parts of the region, making it a suitable and representative setting for this study. Data collection was conducted over a one-month period but included all patients who had undergone colonoscopy between 2017 and 2022.

### Participants and sampling

The study population consisted of all patients who underwent colonoscopy at the Gaza Strip Hospitals during the period 2017–2022. A comprehensive sampling strategy was employed, whereby all eligible patients within the study timeframe were included to maximize representativeness and minimize sampling bias. Patients were identified through the hospital’s medical records system, and data were extracted accordingly. The final sample size comprised 983 patients. Specific exclusion criteria were applied to maintain the study’s focus on colonoscopy-related outcomes:

- Patients who underwent combined double-scope procedures (gastroscopy and colonoscopy simultaneously).
- Patients who underwent gastroscopy alone without colonoscopy.
- Patients who had undergone colonoscopy in years prior to 2017.

### Data collection

**Fig. 1:**
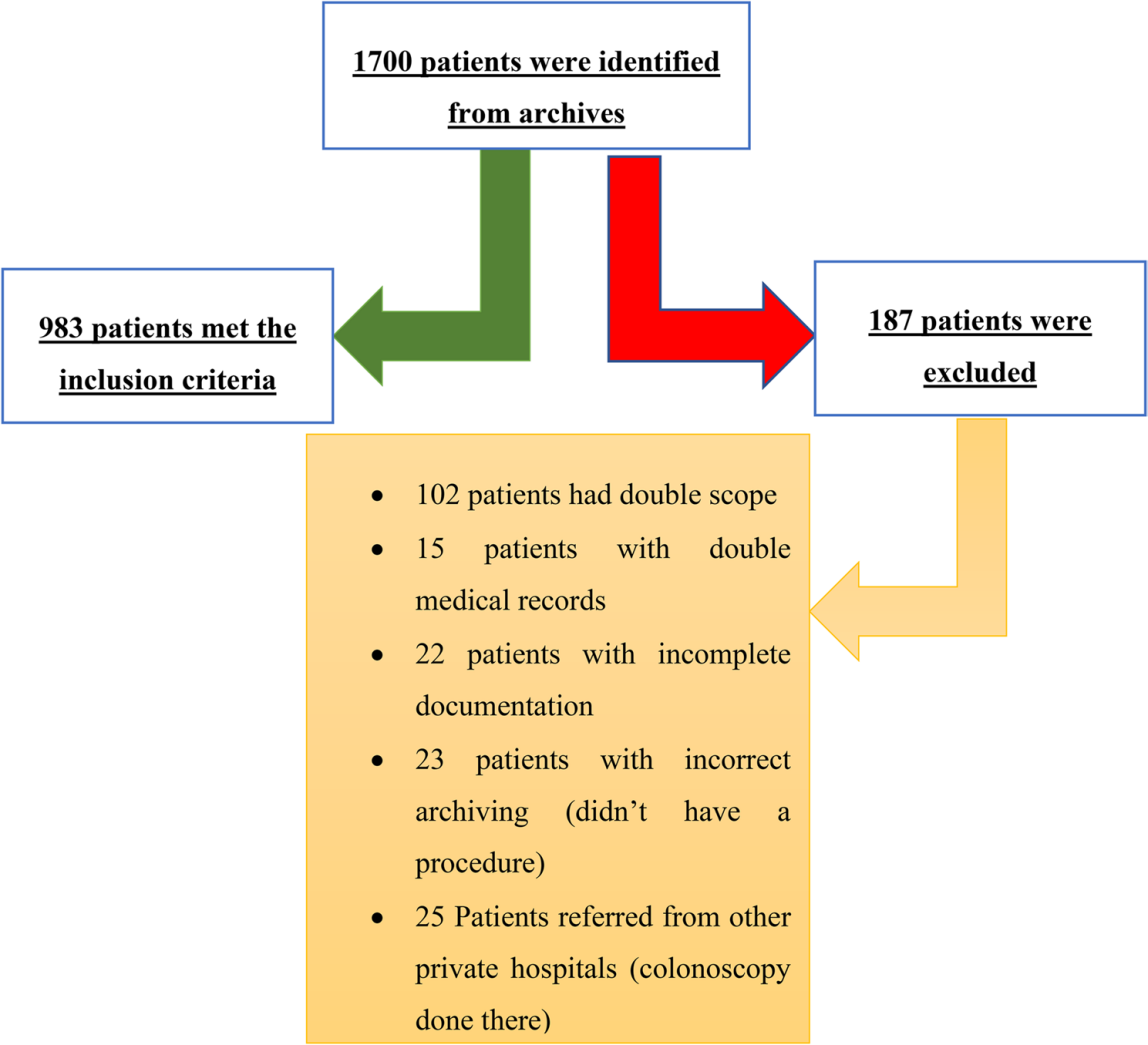
Flow diagram of the process of patients’ selection

Data were retrospectively retrieved from patients’ medical records and entered into a structured questionnaire designed by the researchers. The tool was developed based on an extensive review of recent literature to ensure inclusion of all relevant clinical and demographic variables. The data collection tool included sections covering sociodemographic details (such as age, sex, and residency), presenting complaints, medical history, laboratory investigations, and colonoscopic findings (Supplementary File 1). The retrospective approach ensured that patient management and care were not influenced by the study. Figure 1 presents the process of patients’ selection.

The data collection process was limited by its reliance on medical records, which may contain incomplete, inaccurate, or inconsistently documented information, leading to potential information and measurement bias. Data quality depended on variations in documentation practices across hospitals and clinicians over the 2017–2022 period. In addition, histology reports were kept under different medical records for the patients and that is why histology data were difficult to obtain.

### Study instrument

The primary study instrument was a self-developed structured questionnaire aligned with evidence-based studies and validated tools from recent literature (Supplementary File). It was divided into three main domains:

- Baseline sociodemographic and clinical information.
- Findings and observations documented during the colonoscopy procedure.
- Relevant laboratory investigations conducted prior to or following the procedure.

The instrument was designed to capture complete and accurate data while ensuring clarity and ease of extraction.

### Statistical analysis

Data were analyzed using SPSS version 23. Continuous variables, including age, were summarized using mean, standard deviation, median, and range, while categorical variables were presented as frequencies and percentages. Associations between colonoscopy findings (normal vs. abnormal) and patients’ demographic and clinical characteristics (age group, sex, admission route, and indication for colonoscopy) were examined using the chi-square (χ²) test. Similarly, relationships between procedure-related factors (procedure conclusion, site reached, and type of anesthesia) and physician recommendations were assessed using chi-square analysis. A p-value of < 0.05 was considered statistically significant, and all tests were two-tailed.

### Ethical considerations

Prior to data collection, formal ethical approval was obtained from the Health Research Committee of the Ministry of Health (MoH) in the Gaza Strip. Permission was granted to access patients’ medical records solely for research purposes. The confidentiality and privacy of all patient information were strictly maintained throughout the study. No identifying information was recorded or disclosed, and all data were handled in accordance with ethical principles of medical research and the standards of the Declaration of Helsinki.

## Results

The study included 983 patients who were undergone colonoscopy and were admitted from outpatient department, inpatients or referred from other hospital. The mean age of study participants was 52 *±* 16 years with median age of 53 years. The patients’ age ranged from 13 to 100 years. The male to female ratio was almost 2:1; the study included 604 males (61.4%) and 379 females (38.6%). Most of participants had been admitted through outpatient department schedule (*n* = 920, 93.6%). Table 1 presents demographic characteristics of study participants.

**Table 1:** Demographic characteristics of study participants

Characteristic	Category / Statistic	Frequency (*n*)	Percent (%)
Age groups	< 40	189	19.2
	40–59	428	43.5
	≥ 60	366	37.3
Sex	Male	604	61.4
	Female	379	38.6
Route of admission	Outpatient	920	93.6
	Inpatient / Referral	63	6.4
Year of procedure	2017	156	15.8
	2018	203	20.6
	2019	189	19.2
	2020	30	3.1
	2021	236	24.1
	2022	169	17.2

**Fig. 2:**
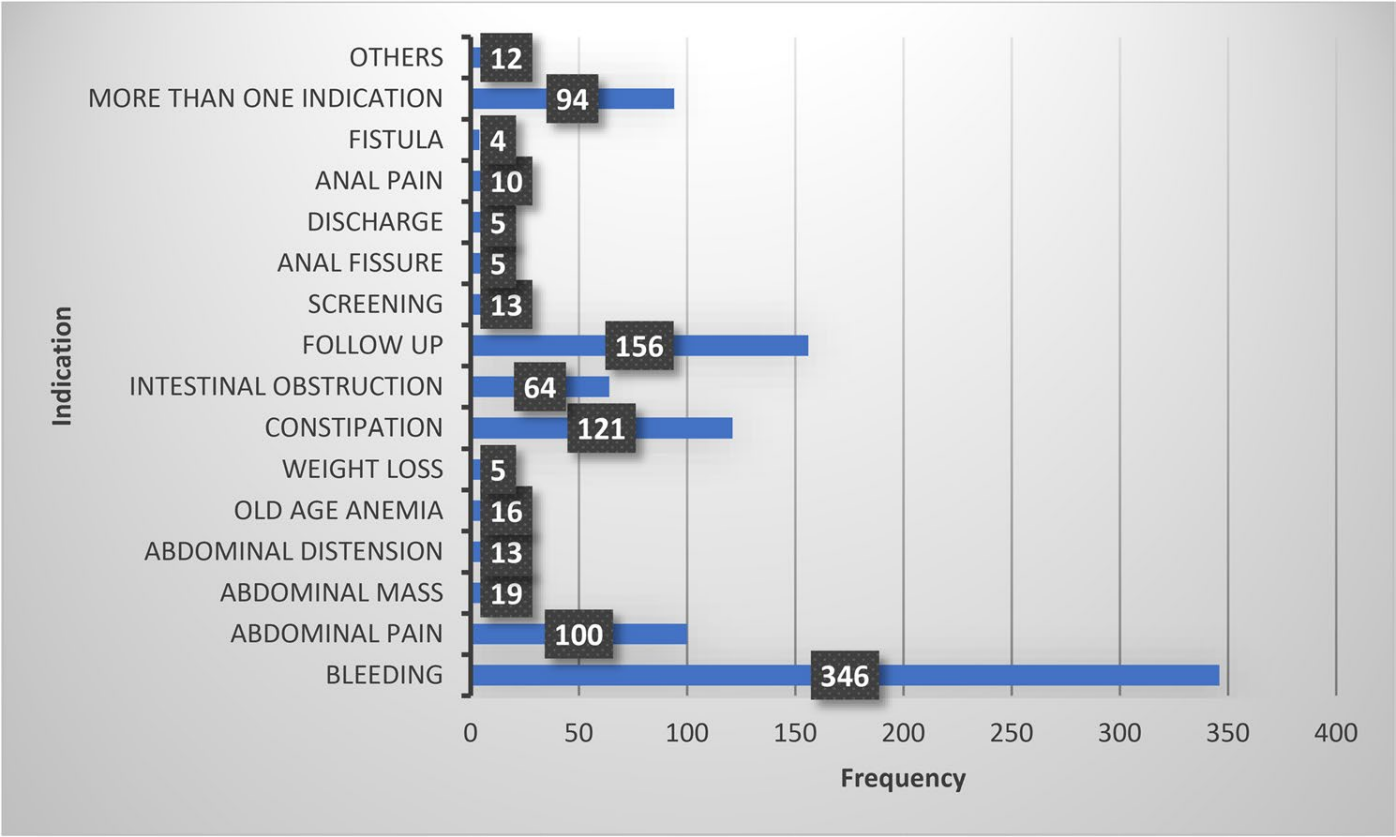
Indication for performing colonoscopy among study participants

The presenting symptom or indication that led to the decision for colonoscopy varied among study participants. The most common symptom was bleeding (*n* = 346, 35.2%) followed by follow up (*n* = 156, 15.9%). Figure 2 shows the frequency of presenting complaint or indication among study participants.

**Fig. 3:**
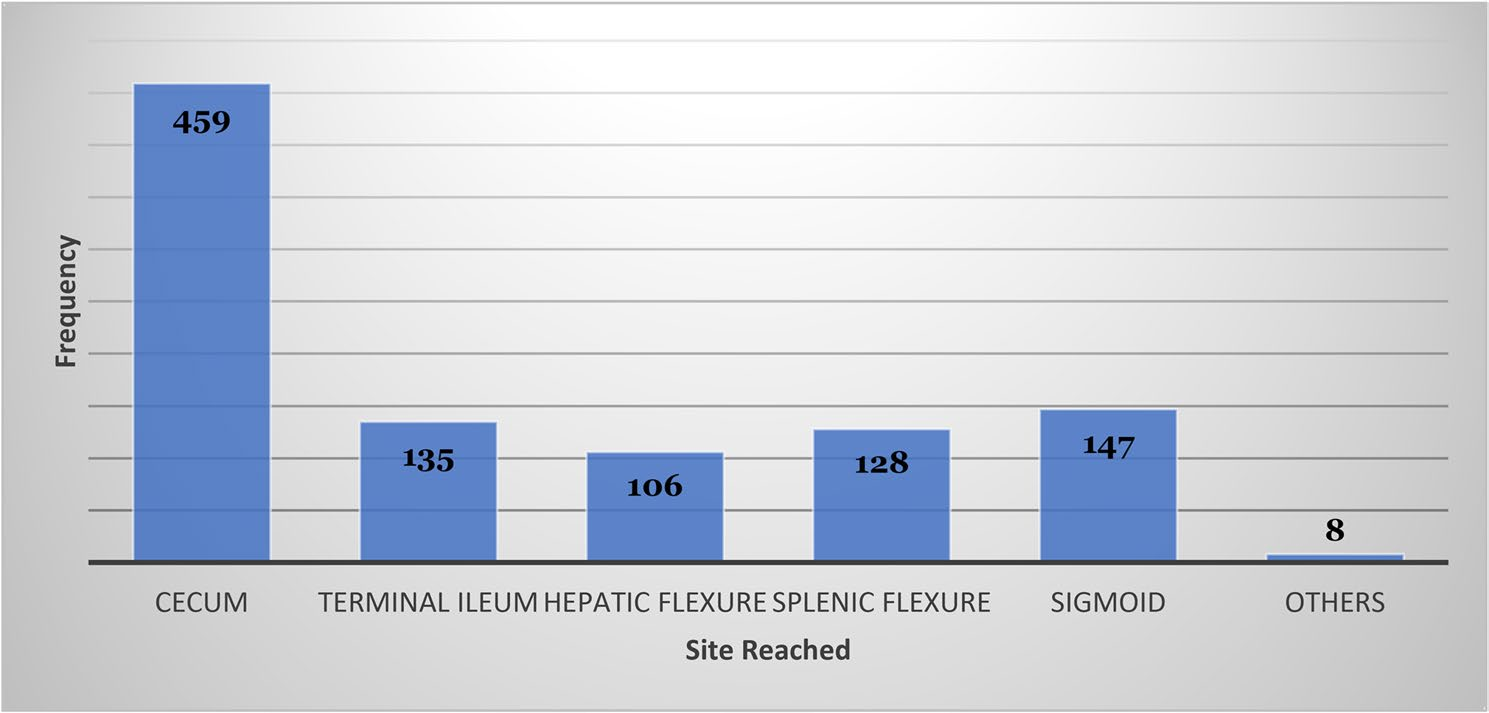
Site reached by colonoscopy procedure

The anesthetic method used for the procedure was either local (*n* = 22, 2.2%) or general anesthesia (*n* = 961, 97.8%). The colonoscopy procedure passed various distances and reached up to terminal ileum. The most frequent site it reached was to cecum (*n* = 459, 46.7%) (Fig. 3).

Table 2 shows that normal colonoscopy findings were the most common outcome, observed in 348 patients (35.4%). Among abnormal findings, masses (11.0%) and polyps (10.2%) were the most frequent, followed by piles (7.5%) and diverticulosis (5.0%). Less common findings included hyperemic mucosa (2.7%), anal fissures (2.2%), ulcers (0.4%), volvulus (0.5%), mucosal thickening (0.5%), fistulas (0.2%), and telangiectasia (0.1%), while other miscellaneous findings accounted for 1.6% of cases.

**Table 2:** Colonoscopy findings among study participants

Colonoscopic findings		Frequency (*n*)	Percent (%)
Normal colonoscopy		348	35.4
Colorectal neoplasia	Colorectal mass / suspected malignant tumor	108	11.0
	Colorectal polyps	100	10.2
Inflammatory and mucosal disorders	Suspicion of UC or Crohn’s disease	27	2.7
	Rectal erythematous mucosa (proctitis-like changes)	10	1.0
	Colonic ulceration	4	0.4
	Mucosal edema / suspected inflammatory changes	5	0.5
Diverticular disease	Diverticulosis	49	5.0
Anorectal disorder	Hemorrhoids (piles)	74	7.5
	Anal fissure	22	2.2
Other structural abnormalities	– Volvulus	5	0.5
	– Fistula	2	0.2
	– Telangiectasia / vascular lesion	1	0.1
	– Poor bowel preparation	23	2.3
	– Other findings	16	1.6

The colonoscopy findings were normal among 350 participants (35.6%). Polyps were found among 95 participants (9.7%) other conclusions were internal piles, rectal mass and diverticulae. The post-procedure recommendations of the physicians performing the procedures varied according to each case; however, some were frequently reported, particularly follow-up (*n* = 374, 38%). Surgical management was recommended for 48 patients (4.9%).

Participants aged 60 years or older demonstrated the highest prevalence of abnormal findings. Male patients and those admitted through non-outpatient routes were more likely to have pathological findings. Bleeding as an indication was strongly associated with abnormal outcomes, whereas follow-up procedures were more often linked to normal findings. Table 3 shows these findings.

**Table 3:** Factors associated with abnormal colonoscopy findings among study participants

Variable	Category	Normal Finding, *n* (%)	Abnormal Finding, *n* (%)	Total	χ²	*p*-value
Age group (years)	< 40	92 (48.7)	97 (51.3)	189	34.2	< 0.001
	40–59	146 (34.1)	282 (65.9)	428		
	≥ 60	110 (29.6)	256 (70.4)	366		
Sex	Male	198 (32.8)	406 (67.2)	604	6.8	0.009
	Female	150 (39.6)	229 (60.4)	379		
Admission route	Outpatient	337 (36.6)	583 (63.4)	920	8.5	0.004
	Inpatient / referral	11 (17.5)	52 (82.5)	63		
Indication	Bleeding	78 (22.5)	268 (77.5)	346	96.7	< 0.001
	Follow-up	92 (59.0)	64 (41.0)	156		
	Other symptoms	178 (36.7)	303 (63.3)	481		

Table 4 demonstrates a strong association between colonoscopy conclusions, procedural characteristics, and physician recommendations (*p* < 0.001). Normal findings were predominantly managed with follow-up alone, whereas masses and other significant abnormalities were more frequently referred for surgical intervention. Procedures reaching the cecum or terminal ileum were associated with a higher proportion of follow-up recommendations, reflecting improved diagnostic completeness. Additionally, general anesthesia was significantly associated with more intensive management pathways, likely reflecting case complexity. These findings highlight how procedural outcomes directly influence clinical decision-making and patient management strategies.

**Table 4:** Association between Procedure-Related Factors, colonoscopy Conclusions, and physician recommendations

Variable	Category	Follow-up *n* (%)	Medical Treatment *n* (%)	Surgical Referral *n* (%)	Total	χ²	*p*-value
Procedure conclusion	Normal	212 (60.6)	138 (39.4)	0 (0.0)	350	284.6	< 0.001
	Polyps	41 (43.2)	32 (33.7)	22 (23.1)	95		
	Mass	19 (17.6)	26 (24.1)	63 (58.3)	108		
	Other findings	102 (23.6)	136 (31.5)	194 (44.9)	432		
Site reached	Rectum–Sigmoid	46 (28.6)	58 (36.0)	57 (35.4)	161	29.3	< 0.001
	Descending/Transverse	104 (32.6)	117 (36.7)	98 (30.7)	319		
	Cecum/Ileum	224 (44.4)	157 (31.2)	123 (24.4)	504		
Anesthesia type	Local	14 (63.6)	8 (36.4)	0 (0.0)	22	11.1	0.004
	General	360 (37.5)	324 (33.7)	277 (28.8)	961		

## Discussion

Colorectal cancer (CRC) remains a significant global health concern, ranking as the third most common malignancy and the second leading cause of cancer-related deaths [1]. In developed countries, widespread implementation of colonoscopy and other screening strategies has contributed to stable or declining CRC incidence rates, particularly among individuals over 50 years [2–5]. However, recent evidence indicates a rising trend of early-onset CRC, with cases increasingly reported in younger populations worldwide [6–9].

In the present study, which included 983 patients undergoing colonoscopy at Gaza Strip hospitals, the mean age was 52 years, and the majority of participants were male. The most frequent indication for colonoscopy was gastrointestinal bleeding, followed by follow-up assessments. These findings are consistent with existing literature that highlights bleeding as one of the most common presenting symptoms of CRC and other colorectal pathologies [8]. Importantly, 35.4% of participants had normal colonoscopic findings, while 19.2% had multiple abnormalities. Significant pathologies included masses (11.0%) and polyps (10.2%), both of which are known precursors or indicators of colorectal malignancy. This reflects global observations that colonoscopy is an invaluable diagnostic and preventive tool for identifying neoplastic and pre-neoplastic lesions before progression to advanced disease [3, 7].

Our findings also align with regional reports suggesting that CRC often presents at a younger age in Middle Eastern populations compared to Western counterparts [12–14]. Similar to trends observed in Saudi Arabia, where the mean age of CRC diagnosis is 12–15 years earlier than in Western countries [12–15], the relatively young median age in our cohort (53 years) underscores the need for heightened clinical vigilance in this demographic. Although our study was not limited to CRC cases, the detection of masses and polyps in a substantial proportion of patients supports the call for earlier and more accessible screening programs.

Previous studies have shown that younger CRC patients are more likely to present with advanced-stage disease, often due to diagnostic delays and limited suspicion of malignancy in this age group [8, 9]. While staging was not available in our dataset, the detection of obstructive masses and the need for surgical referrals in nearly 5% of cases emphasize the role of colonoscopy in facilitating timely intervention. Flexible sigmoidoscopy has been suggested as a cost-effective alternative for symptomatic younger patients, particularly in low-resource settings, where colonoscopy access may be limited [16–22]. However, given that our findings included pathologies throughout the colon, a complete colonoscopy remains the preferred diagnostic tool whenever feasible.

The increasing incidence of early-onset CRC reported in international registries [23]– [24] and the demonstrated effectiveness of colonoscopy in reducing both incidence and mortality in patients aged ≥ 50 years [24] have prompted revisions to screening guidelines. The American Cancer Society now recommends average-risk CRC screening beginning at 45 years of age [4]. In comparison, many low- and middle-income regions, including the Gaza Strip, lack comprehensive population-based screening programs. Our study highlights the urgent need to strengthen endoscopy services and establish structured screening initiatives tailored to local demographics and healthcare capacities.

In the present study, diverticulae were detected in 5.0% of participants, which represents a relatively small proportion compared to reports from Western populations [25], where the prevalence of colonic diverticulosis is higher, particularly in older adults. This observation aligns with data from a prospective screening colonoscopy study, where 27.2% of patients were found to have incidental diverticulosis, predominantly in the left colon, and the long-term risk of developing clinically significant diverticular disease was low, with only 4.2% of patients experiencing diverticulitis or diverticular bleeding over a mean follow-up of seven years. Similarly, in our cohort, most cases of diverticulae were asymptomatic and identified incidentally during colonoscopy for other indications, such as bleeding or follow-up evaluation.

The relatively low rate of inadequate bowel preparation in our cohort suggests generally good patient compliance and may reflect dietary patterns rich in fiber, which could also contribute to the observed prevalence of diverticular disease. Regional lifestyle factors may further influence colorectal pathology, including traditionally low alcohol consumption and relatively limited red meat intake, which are considered protective against colorectal cancer, contrasted with high smoking rates that may increase risk. Despite the presence of several potentially protective environmental factors, the notable frequency of advanced endoscopic findings in this study implies that patients often present at later stages of disease, likely due to limited access to preventive screening and delayed healthcare seeking. Although this study cannot establish causality, framing these findings within the regional context highlights important gaps in early detection and reinforces the relevance of strengthening screening and diagnostic capacity in Gaza.

Although the retrospective design and absence of histopathological confirmation limit causal inference and prevent direct comparison with screening-based colonoscopy studies, the present findings remain clinically informative when interpreted in context. The proportion of mass lesions observed in our cohort is substantially higher than rates typically reported in screening populations (< 1%), and is more consistent with studies of symptomatic patients presenting with hematochezia, where abnormal findings have been reported in 8–40% of cases [26]– [27]. This pattern likely reflects delayed presentation and the predominance of symptom-driven referrals rather than preventive screening in Gaza. Beyond describing procedural outcomes, this study provides a “satellite view” of current endoscopy practice and diagnostic yield in a resource-constrained setting, highlighting both existing service capacity and the apparent burden of advanced pathology. Collectively, these findings underscore the potential public health value of expanding access to organized colorectal screening and earlier diagnostic pathways in the Gaza Strip.

These findings underscore the generally favorable natural history of incidental diverticulosis, suggesting that, in resource-limited settings like the Gaza Strip, incidental diverticulae may not require immediate intervention but should be documented for longitudinal monitoring. Moreover, the low prevalence of diverticular disease in our population may reflect demographic factors, dietary habits, and the limited use of routine screening colonoscopies, highlighting the need for increased endoscopic capacity to better understand and manage colonic diverticular conditions in this region.

Limitations of this study include its retrospective design and restriction to a single regional setting, which may limit generalizability. Nonetheless, with nearly 1,000 patients over a 5-year period, the study provides valuable insights into the indications, findings, and outcomes of colonoscopy in the Gaza Strip.

## Conclusion

In this retrospective analysis of 983 patients undergoing colonoscopy in Gaza Strip hospitals, the study population was predominantly middle-aged with a male predominance, and most procedures were performed on an outpatient basis. Bleeding was the most frequent indication for colonoscopy, and the cecum was the most commonly reached anatomical site, reflecting adequate procedural completeness in the majority of cases. Although over one-third of examinations yielded normal findings, a substantial proportion of patients demonstrated abnormal or multiple findings, including polyps, masses, diverticulae, and other pathological conditions. Inferential analyses revealed that abnormal colonoscopy findings were significantly associated with older age, male sex, inpatient or referral admission, and bleeding as the presenting indication. Furthermore, colonoscopy conclusions were strongly linked to subsequent clinical management, with normal findings largely resulting in follow-up recommendations, while significant abnormalities—particularly masses—were more frequently associated with surgical referral. Overall, these results highlight the diagnostic value of colonoscopy in identifying clinically relevant pathology and guiding patient management, particularly among higher-risk patient groups and those presenting with alarm symptoms.

## Supplementary Information

Supplementary Material 1.

Supplementary Material 2.

## Data Availability

Data are available upon reasonable request.

## Abbreviations

CRC: Colorectal cancer
MoH: Ministry of health
SEER: Surveillance, epidemiology, and end results
SPSS: Statistical package for the social sciences
UC: Ulcerative colitis
