# Supplementary material for: Characteristics and outcomes of patients undergoing colonoscopy in Gaza Strip hospitals: a retrospective study
Source: BMC Gastroenterol. 2026 Feb 6;26:159. doi: 10.1186/s12876-026-04673-0 (PMC12973562; doi:10.1186/s12876-026-04673-0)
Supplement: Supplementary file 1 — Supplementary Material 1. [file 12876_2026_4673_MOESM1_ESM.docx]

**Characteristics and Outcomes of Patients Undergoing Colonoscopy in Gaza Strip Hospitals: A Retrospective Study**

**Section A: Demographic Data**

A1. Gender (Tick one)

☐ Male (1)

☐ Female (2)

Age: _______

**Section B: Clinical Indications for Colonoscopy (Tick all that apply)**

☐ Bleeding (1)

☐ Abdominal pain (2)

☐ Abdominal mass (3)

☐ Abdominal distension (4)

☐ Old age anemia (5)

☐ Weight loss (6)

☐ Constipation (7)

☐ Intestinal obstruction (8)

☐ Follow-up (9)

☐ For screening (10)

☐ Anal fissure (11)

☐ Discharge (12)

☐ Anal pain (13)

☐ Fistula (14)

☐ Others (15): __________________________

**Section C: Admission Status**

☐ OPD (1)

☐ IPD (2)

☐ Referred from other hospitals (3)

**Section D: Anesthesia Used During Colonoscopy**

☐ General anesthesia (1)

☐ Local spray (2)

**Section E: Extent of Scope Passage**

☐ Up to cecum (1)

☐ To terminal ileum (2)

☐ To hepatic flexure (3)

☐ To splenic flexure (4)

☐ To sigmoid (5)

☐ Others (6): __________________________

**Section F: Colonoscopy Findings (Tick all that apply)**

☐ Normal (1)

☐ Hyperemic mucosa (colitis) (2)

☐ Diverticulae (3)

☐ Mass (4)

☐ Polyp (5)

☐ Telangiectasia (6)

☐ Bad preparation (7)

☐ Ulcer (8)

☐ Piles (9)

☐ Hyperemic mucosa of the rectum (10)

☐ Anal fissure (11)

☐ Volvulus (12)

☐ Thickening of mucosa (13)

☐ Fistula (14)

☐ Others (15): __________________________

**Section G: Final Colonoscopic Conclusion (Tick one or more as appropriate)**

☐ Normal (1)

☐ Internal piles (2)

☐ Bad preparation (3)

☐ Proctitis (4)

☐ Colonic mass (5)

☐ Rectal mass (6)

☐ Polyp (7)

☐ Colitis (8)

☐ Diverticulae (9)

☐ Anal fissure (10)

☐ Intestinal obstruction (11)

☐ Thickening of mucosa (12)

☐ Fistula (13)

☐ Others (14): __________________________

**Section H: Recommendations After Colonoscopy (Tick all that apply)**

☐ Follow-up (1)

☐ Further testing (2)

☐ Return to treating doctor (3)

☐ Antibiotics (4)

☐ Surgical treatment (5)

☐ Laxatives (6)

☐ MDT discussion (7)

☐ Others (8): __________________________

Patient Record Number: _____________ Date of Colonoscopy: ____ / ____ / ______

Hospital Name: _______________________________
